# Regulation of Marriage to Cut down Ratio of Insanity

**Published:** 1910-04

**Authors:** 


### TEXAS MEDICAL JOURNAL.

Established July, 1885

F. E. DANIEL, M. D., - - - - - - Editor, Publisher and Proprietor

Published Monthly.—Subscription $1.00 a Year.

VOL. XXV AUSTIN, APRIL, 1910. No. 10

The publisher is not responsible for the views of the contributors.

### Original Articles.

For Texas Medical Journal.

### Regulation of Marriage to Cut Down Ratio of Insanity.

(Reproduced from the *Houston Chronicle,* March 21.)

Calling attention to the menace of alcoholism in general and its effect on the nation, Dr. Charles L. Gregory, Superintendent of the North Texas Hospital for the Insane, before a large audience at the Beach Auditorium on Sunday afternoon, suggested a startling method for the protection of the generation to come, the sterilization of the inebriate and the imbecile, and cited the approval of the most eminent medical authorities of the day to give force to the proposition.

Dr. Gregory delivered three lectures in the city on Sunday, one in the morning at the McKee Street Methodist Church, one in the evening at Fraternal Hall, Houston Heights, and the third in the afternoon at the Beach Auditorium. The afternoon lecture was given before an audience which largely appreciated the startling statistics which Dr. Gregory had to offer on the increase of alcoholic insanity in the United States, and the cure he had to suggest. The text of the afternoon lecture follows:

I wish to call your attention to the effects of alcohol generally, said Dr. Gregory, and to show why the physician should disclose these effects. The physician should be, as he is, the greatest enemy of alcohol, because he, more than any other, comes in contact with its disastrous effects. The medical profession knows very well that it is a patent cause of disease, degeneracy, crime and death. It is not a real stimulant, it does not give strength to the mental powers, but it paralyzes the regulative apparatus of the mind, and hence the person under its influence is not able to appreciate his real condition. It is a matter of common knowledge that a person under the primary influence of alcohol is very conversant, more witty, more social, more generous in sentiment, but he is not so careful in his statements, and has not a proper appreciation of his own position or that of others while under its influence. He loses self-control, self-respect, self-restraint, and the sense of responsibility and the power of judging between right and wrong. In a word I wish to suggest that it is generally recognized by the leading physicians of this country and Europe that alcohol dethrones the highest functions of the brain and leaves it in a depressed condition dependent upon the reserve forces of nature to restore it.

It is my purpose in this lecture to deal with alcoholic insanity. It is difficult, however, to avoid repetition as each phase of the subject possesses so much in common with the others that each discussion seems to dovetail into the other. "Like causes produce like effects," so in each case our base is the same, the effects of alcohol. But for convenience and clearness we present the subject under different headings.

Alcoholic insanity may be divided into three groups: (1) "Acute Alcoholic Mania." (2) "Delirium Tremens." (3) "Chronic Alcoholic Dementia."

In acute alcoholic mania and delirium tremens the brain cells of the motor centers are greatly irritated, which is evidenced in muscular excitement and uncontrolled movements. Men suffering from these troubles develop hallucinations of hearing and sight. They hear voices which to them are clear and often excite them to actions of destruction. The illusions of sight are so marked that figures of friends are not recognized. A dark place on the wall may excite an insane belief which it is impossible for relations or friends to dispel, and which may render the patient dangerous to others.

In acute mania the patient becomes furious. The whole brain is in a turmoil and is completely paralyzed as to its normal power of understanding. We have seen young men in the prime of life suffering from outbreaks of insanity following the excessive use of alcohol. A large per cent of these cases recover in four or six weeks under scientific treatment, but relapse upon resorting to alcohol. These patients would not come to an asylum had not many of them inherited an irresistible desire to drink.

Delirium tremens follows repeated debauches. The marked depression under which the nervous system labors is often obscured by restlessness, gloominess and timidity of the patient. He is incapacitated to think or make simple decisions. He hears voices, mocking demons, ghosts and evil beasts, sees snakes and scorpions and is frequently pursued by tormenting objects. This condition is due to the fact that the sense centers of the cerebrum are perverted from overstimulation. The centers of judgment, reason and decision are poisoned and the bravery an ordinary man would exhibit is wanting. The patient cowers and cringes before terrors resulting from a wrongly acting brain. The centers governing motives are upset in a temporarily insane person, a condition often seen in victims of alcohol struggling with those who attempt to control and succor them.

Alcoholic dementia comes on slowly as a result of strong drink. Mental deterioration is observed in lack of ability to deal with facts in an intelligent way. The patient is unable to rightly appreciate his condition, hence he disregards the advice of friends and loved ones. He is easily offended, exhibits insane delusions of suspicion and jealousy leading to unfounded charges being made against others. The wife suffers mental anguish on account of false charges circulated by her husband while in this condition. These outbursts of emotional insanity are due to the loss of higher intellectual control. Slowly the symptoms of premature senility of mind appear, the moral sense becomes blunted, social feelings and affections disappear. The demoralization appears in selfish, brutish and indecent acts.

### INSANITY INCREASES.

"Alcoholic insanity goes steadily up. This year no less than 42.3 per cent of all our men and 18 per cent of our women—much the largest proportion we have ever had experience of—had excess in alcohol assigned as the cause of their insanity. No explanation will account for this, but that certain classes of our population are drinking to greater excess than they did. And in so doing many of them are destroying their sanity." This is Dr. Clouston's report of the Morning Side Asylum, 1903.

Alcoholism has propagated its influence and has spread its roots and fibers of destruction through the blood soil of children's children until we have multiplied thousands of physical and mental degenerates who will become public charges. Last year on account of defective physical development this country witnessed the most appalling death rate in children under five years of age that it has witnessed during its history.

### REGULATION OF MARRIAGE.

I claim that every child has a right to be born healthy and not marked by disease before birth through the sins of its parents. Marriage is not regarded as it should be; the law troubles itself less about marriage than anything which it governs. The law should make marriage more difficult so as to prevent evil effects of drinking and heredity. Stock breeders are more careful in breeding animals than we are in safeguarding the human race.

"In ancient and modern Hebrew history child creation was a prime consideration." And today it should be of first importance with us. Not only should insane people, habitual drunkards, criminals, epileptics and consumptives be debarred from marriage, but children should be disallowed the privilege of marriage. Oliver Cromwell raised the age of marriage in England, yet we are permitting our children to marry at the age of 14 and 15. As long as we indorse the marriage of the above classes, lunatics, criminals and prostitutes will abound in this country. If the aforesaid classes desire to marry they should be sterilized under the direction of a suitable commission of physicians. With the insane and criminal vasectomy should be compulsory. Sterilization of the defective has been adopted by several States in the Union and Texas should fall in line. The *Journal* of the American Medical Association recommends sterilization of the insane and criminals, as does the Chicago Physicians' Club, the Southern District Medical Society and the Chicago Society of Hygiene.

When the people are educated along these lines, when they are convinced that it is not inhuman to sterilize defectives, they will demand the enactment of such wholesome laws. Of course no measure ought to be adopted unless it is approved and supported by public opinion, as it would prove ineffective. That the people should be educated as to the causes of so many defectives throughout the country no man will gainsay.

### STARTLING STATISTICS.

In 95 per cent of the homicides of Germany the guilty person is brought to justice. In Spain the number of convictions is 85 per cent of the total number of crimes. In France it is 61 per cent, in Italy 77 per cent, in England 50 per cent. These unchallenged facts when offset against our two convictions in every

100 murders explains why drunkenness, lawlessness and murder are increasing by leaps and bounds in the United States. "In 1908 the United States had more murders than Italy, Austria, France, Belgium, England, Ireland, Scotland, Spain, Hungary, Holland and Germany combined."

### AGAINST ALCOHOLISM.

I assert that alcohol should die for the following reasons:

1. Alcoholism readily passes into unmistakable insanity, and it is always a cause of nervous degeneracy in the children born within its influence.

2. A small per cent of the children of drunkards are mentally sound, while a large per cent are mentally weak.

3. Children of alcoholics die at an early age, and the families of drunkards seldom extend beyond four generations.

4. Drunkards cause to be born into the world criminals, imbeciles, idiots, degenerates and lunatics.

5. It causes a degeneration of nerve centers and impairs nerve cells.

6. It is in a large measure responsible for every larceny, rape and murder committed in this country.

7. It produces a wasting of the heart muscles and a fatty degeneration of the liver and kidneys.

8. It causes a diseased or hardened condition of the blood-vessels and as a result of this we have poor nourishment and faulty elimination of the refuse products of the body. Hence the blood can not supply oxygen and carry off poison.

9. It depresses the activity of organs and affects the brain, especially the pyramidal brain cells which are the physical instruments of thought, will and memory, and the means of communication between the mind and the outer world. Thus the exercise of these important spiritual faculties is retarded and inhibited and as a result of the foregoing we have impulsive, degenerative insanity.

### WORLD IS BETTER.

The world is growing better, but not where the mighty statistics of the tobacco, liquor and fashion trades find their surest foundations. The world is not growing better at those particular points where alcohol in all its forms breeds the degeneration and death implied in our unchallenged catalogue of crime, disease and misery.

The doctors know very well about the "tobacco heart," the

"whisky liver," the "whisky kidney," brain and nerves; they know of "tobacco blindness." And the man who in the face of all this persists in his cigar and encourages by example, if not by precept, the degeneracy of his son in the cigarette is not helping the world to grow better.

It has been my purpose, in the foregoing lecture, to give facts. Consequently I have given more attention to accuracy of statement than to ornament. My desire is to help the rising generations, to place before the youth of my country object lessons that will call attention to the terrible consequence of bad habits, and to point out the penalties they must surely pay for violated law.

